# Enhancing clinical diagnosis of laryngeal cancer through fusion-based transfer learning with Osprey Optimisation Algorithm using histology images

**DOI:** 10.3389/fonc.2025.1618349

**Published:** 2025-10-08

**Authors:** Nouf Al-Kahtani, Mona M. Jamjoom, Mohamad Khairi Ishak, Samih M. Mostafa

**Affiliations:** ^1^ Department of Health Information Management and Technology, College of Public Health, Imam Abdulrahman bin Faisal University, Dammam, Saudi Arabia; ^2^ Department of Computer Sciences, College of Computer and Information Sciences, Princess Nourah Bint Abdulrahman University, Riyadh, Saudi Arabia; ^3^ Department of Electrical and Computer Engineering, College of Engineering and Information Technology, Ajman University, Ajman, United Arab Emirates; ^4^ Computer Science Department, Faculty of Computers and Information, South Valley University, Qena, Egypt

**Keywords:** laryngeal cancer, histology images, fusion transfer learning, clinical diagnosis, Osprey Optimisation Algorithm

## Abstract

**Background:**

Laryngeal squamous cell carcinoma is the most commonly diagnosed neck and head cancer. In contrast, the primary stage of pre-malignant and laryngeal cancer (LC) has to be handled with early diagnosis and treated with higher levels of laryngeal protection. Radiological evaluation with magnetic resonance imaging (MRI) and computed tomography (CT) techniques offers essential information on the disease in terms of the distance of the principal cancer and the existence of cervical lymph node metastasis. Recently, numerous deep learning (DL) and machine learning (ML) models have been implemented to classify the extracted features as either cancerous or healthy.

**Methods:**

In this study, the Clinical Diagnosis of Laryngeal Cancer via Histology Images using the Fusion Transfer Learning and the Osprey Optimisation Algorithm (CDLCHI-FTLOOA) model is proposed. The aim is to improve the LC detection outcomes using histology image analysis to improve the patient’s life. Initially, the CDLCHI-FTLOOA model utilizes median filtering (MF)-based noise elimination during the image pre-processing process. Furthermore, the feature extraction process is performed by using the fusion models, namely AlexNet, SqueezNet, and CapsNet. The autoencoder (AE) method is employed for classification. To improve model performance, the Osprey Optimisation Algorithm (OOA) method is used for hyperparameter tuning to choose the optimal parameters for improved accuracy.

**Results:**

To exhibit the enhanced performance of the CDLCHI-FTLOOA model, a comprehensive experimental analysis is conducted under the laryngeal dataset. The comparison study of the CDLCHI-FTLOOA model portrayed a superior accuracy value of 97.16% over existing techniques.

**Conclusion:**

Therefore, the proposed model can be employed for the accurate detection of the LC using the histopathological images.

## 1 Introduction

Laryngeal cancer accounts for approximately 2% of all cancers globally and is thought to be one of the most aggressive types of head and neck cancer. The risk factors for laryngeal tumor growth include alcohol consumption, smoking, occupational substances, polluted environment, and heredity (1). The outcome for the Laryngeal tumor diagnosis is decided based on certain aspects like the cancer’s stage, grade, the concerned place in the larynx, the patient’s lifestyle, and well-being after the initial analysis. Both initial and precise analyses are crucial for initiating appropriate treatment and extending the patient’s lifespan (2). Radiological analysis with MRI and CT provides significant information about the cancer level and the occurrence of cervical lymph node metastasis; however, it fails to recognize the superficial mucosal irregularities. Currently, white light laryngoscopy with biopsy is regarded as the gold standard for diagnosing LC and precancerous lesions. However, it generates poor-quality images and suffers from problems in classifying minute epithelial variations and distinguishing benign tumors from malignant ones.

Narrow band imaging (NBI) is an endoscopic imaging procedure aimed to diagnose mucosal lesions of the larynx which are invisible in white-light endoscopy, but are distinctive of pre-tumor and tumor lesions of the larynx (3). NBI endoscopy is an optical approach which enables in enhancing the detection of laryngeal lesions, carry out a limited a controlled perioperative biopsy, and refines the clinical scope. The NBI endoscopy is an appropriate approach to identify larynx cancerous lesions at the early state. Recently, the Orbeye™ is commonly utilized in neurosurgery; but, its likely in traditional open surgery has not yet been fully used. Because of its magnification capacity, the Orbeye™ exoscope is a valued tool for assisting surgeons detect and hold the integrity of the recurring laryngeal nerves and parathyroids in the thyroid surgery (4).

After diagnosing the patients with initial-phase cancers, organ preservation-based surgical techniques that require the maximum tumor removal while preserving normal tissues are carried out so that the patient can gain health benefits (5). However, it is challenging to secure a resection margin due to the complex anatomical larynx structures, while the choices concerning the amount of resection are also vital in this regard. In general, the histological changes between the squamous and healthy cell carcinoma tissues are noticeable. However, it is difficult to differentiate the tissues by examining them with the naked eye, even though by visuals like narrow-band imaging, specifically in cancer limits (6). At present, the typical intraoperative diagnosis by the Hematoxylin and Eosin (H&E) staining process involves a series of lengthy steps, namely staining, freezing, and sectioning. Additionally, the process demands seasoned professionals to conduct the intraoperative analysis (7).

So, the medical process becomes complicated and produces inconsistencies in the results generated by different pathologists. In this background, the imaging devices that create accurate and rapid descriptions of the usual and neoplastic tissues are essential. In literature, the authors attempted to measure the LC patient’s survival through computer-aided image analysis approaches, utilizing H&E-stained microscopy images (8). In general, pathologists require long-term training, while the availability of skilled histopathologists is limited. On the other hand, in recent years, the convolutional neural networks (CNNs) method has been established to have the potential to diagnose diseases with high accuracy in less time than healthcare professionals. Thus, this method might support the diagnosis of LC in its early stages (9). DL, a type of ML technique, functions based on the neural network (NN) technique across multiple data formats. The DL-aided methods have been proven to manage both classification and detection problems. In literature (10), an artificial intelligence (AI)-based deep CNN (DCNN) model was utilized to diagnose the Laryngeal tumors through histology images. With this cutting-edge DL approach, the AI technique may directly provide a precise analysis using the image data, which will help in identifying the disease in its early stages and, in turn, increase the survival rate of the patient. LC pose a critical health hazard due to its aggressive nature and the threats involved in early and accurate detection. Conventional imaging techniques often struggle to detect subtle tissue anomalies that may delay diagnosis and treatment. Improving diagnostic precision is crucial for tailoring treatment strategies effectively and improving patient survival rates. Employing advanced computational methods for analyzing histology images can provide deeper insights into tumor characteristics. This can facilitate timely intervention and better management of the disease, ultimately improving clinical outcomes.

In this study, the Clinical Diagnosis of Laryngeal Cancer via Histology Images using the Fusion Transfer Learning and the Osprey Optimisation Algorithm (CDLCHI-FTLOOA) model is proposed. The aim is to improve the LC detection outcomes using histology image analysis to improve the patient’s life. Initially, the CDLCHI-FTLOOA model utilizes median filtering (MF)-based noise elimination during the image pre-processing process. Furthermore, the feature extraction process is performed by using the fusion models, namely AlexNet, SqueezNet, and CapsNet. The autoencoder (AE) method is employed for classification. To improve model performance, the Osprey Optimisation Algorithm (OOA) method is used for hyperparameter tuning to choose the optimal parameters for improved accuracy. To exhibit the enhanced performance of the CDLCHI-FTLOOA model, a comprehensive experimental analysis is conducted under the laryngeal dataset. The key contribution of the CDLCHI-FTLOOA model is listed below.

- The CDLCHI-FTLOOA approach integrates an MF-based image pre-processing stage to suppress salt-and-pepper noise and improve image quality. This enhances the visibility of critical structural patterns within throat region images. Preserving significant anatomical details ensures more accurate feature extraction. This step significantly contributes to the robustness of the overall diagnostic process.
- The CDLCHI-FTLOOA technique incorporates AlexNet, SqueezeNet, and CapsNet in a fusion framework for extracting multiscale and diverse features from throat images. This hybrid extraction improves the capability of the model in distinguishing subtle discrepancies in tissue characteristics. The fusion model also strengthens the representation of both low-level textures and high-level abstractions. This comprehensive feature set improves classification accuracy between cancerous and non-cancerous regions.
- The CDLCHI-FTLOOA methodology combines an AE-based classifier for effectively compressing and reconstructing the extracted features, enhancing the learning process. This model also mitigates feature dimensionality while retaining critical data, resulting in more efficient training. It improves the capability of the technique in generalizing from intrinsic data. The model also attains a more accurate classification of LC in throat region images.
- The CDLCHI-FTLOOA model implements the OOA technique for fine-tuning the hyperparameters of the AE classifier, improving its overall performance. This optimization improves model accuracy by effectually searching the parameter space. It also accelerates convergence during training, mitigating computational time. As a result, the model achieves more reliable and precise LC classification.
- The novelty of the CDLCHI-FTLOOA method is in its integration of three distinct CNN models, such as AlexNet, SqueezeNet, and CapsNet, with an AE classifier, creating a robust and diverse feature representation. This hybrid approach effectually captures multiscale and intrinsic features more effectively than single models. Moreover, the integration of a biologically inspired OOA for tuning additionally refines the learning capability of the model. Altogether, these components synergistically improve classification accuracy and robustness for LC detection.

## 2 Literature survey

In literature (11), a DL-aided method called SRE-YOLO was developed to provide instant support for less-skilled workers in laryngeal diagnosis by spontaneously identifying the lesions using various measures with Narrow-Band Imaging (NBI) and endoscopic White Light (WL) images. The existing methods encounter difficulties in diagnosing new types of lesions. At the same time, there exists a necessity for accurate classification of the lesions to follow a suitable disease management protocol. Traditional diagnostic procedures heavily rely upon endoscopic analysis that frequently needs seasoned professionals to execute the diagnosis, and the outcomes may suffer from bias. Meer et al. (12) developed a complete automatic framework named Self-Attention CNN and Residual Network information optimizer and fusion. The expansion procedures were executed during testing and training examples, while dual progressive deeper methods were also trained. Self-attention MobileNet-V2 techniques were introduced in this study and validated using an augmented dataset. Simultaneously, the Self-Attention DarkNet-19 methods were taught using similar datasets, while the hyperparameters were fine-tuned using the whale optimization algorithm (WOA). Albekairi et al. (13) suggested the temporal decomposition network (TDN), a fresh DL approach that enhances the multimodal medical image fusion process through adversarial learning mechanisms and feature-level temporal examination. The TDN framework integrates two essential modules: a productive adversarial system for temporal feature matching and a salient perception system to discriminate the feature extraction process. The salient perception system classifies and identifies different pixel distributions over diverse imaging modalities, while the adversarial module enables precise feature mapping and fusion. Joseph et al. (14) devised a primary laryngeal tumor classification model by integrating handmade and deep features (DF). By utilizing the handcrafted and transfer learning (TL) features and by deploying the first-order statistics (STAT) and local binary patterns (LBP), the DenseNet 201 was removed in larynx endoscopic narrow-band imaging and fusing, thus resulting in the production of many illustrative features. After hybridizing the features, the best ones were selected using recursive feature elimination with the RF (RFE- RF) model.

Ahmad et al. (15) suggested a fresh, novel attention-based technique named MANS-Net that used spatial, channel, and transformer-based attention models for addressing the issues mentioned earlier. The MANS-Net model effectively studied rough, spatial, color-based, and granular features and increased the nuclei segmentation. The DL techniques were suggested to deliver solutions for the medical tasks. Alrowais et al. (5) presented a novel Laryngeal Cancer Classification and Detection in which the Aquila Optimisation Algorithm was deployed along with the DL (LCDC-AOADL) technique for the classification of neck area images. This technique aimed at inspecting the histopathologic images for both classification and recognition of the Laryngeal tumors. In this approach, the Inceptionv3 method was utilized to extract the features. Also, the LCDC-AOADL approach used the DBN technique to identify and classify the LC. Krishna et al. (16) introduced a different explainable decision-making system utilizing CNN through an intelligent attention mechanism. This mechanism leveraged the response-based feed-forward graphical justification method. In this study, diverse DarkNet19 CNN methods were used to identify the histopathology images. To enhance visual processing and improve the performance of the DarkNet19 model, an attention branch was combined with the DarkNet19 model, thus creating an attention branch network (ABN). In literature (17), specific DL approaches are proposed for nuclei segmentation. Nevertheless, such techniques seldom resolved the issues mentioned earlier. Further, information regarding the problems encountered in H&E-stained histology images is available in public space for reference, while the rest of the data is within the spatial region. Several issues can be resolved by taking spatial and channel features simultaneously. Hu et al. (18) evaluated the efficiency of AI integrated with flexible nasal endoscopy and optical biopsy techniques such as narrow band imaging (NBI), Storz professional image enhancement system (SPIES), and intelligent spectral imaging color enhancement (ISCAN) for early and accurate detection of LC. Alzakari et al. (19) introduced an automated Laryngeal Cancer Diagnosis using the Dandelion Optimiser Algorithm with Ensemble Learning (LCD-DOAEL) methodology by integrating Gaussian filtering (GF), MobileNetV2 for feature extraction, DOA for hyperparameter tuning, and an ensemble of BiLSTM, extreme learning machine (ELM), and backpropagation neural network (BPNN) techniques for accurate classification of throat region images.

Al Khulayf et al. (20) developed a Fusion of Efficient TL Models with Pelican Optimisation for Accurate Laryngeal Cancer Detection and Classification (FETLM-POALCDC) methodology for improving automatic and precise detection of laryngeal cancer using advanced image pre-processing, DL feature fusion, and optimized classification. Sachane and Patil (21) proposed a hybrid Quantum Dilated Convolutional Neural Network–Deep Neuro-Fuzzy Network (QDCNN-DNFN) technique within a federated learning (FL) model to enable early and accurate detection of LC using both image and voice data. Xie et al. (22) proposed a multiparametric magnetic resonance imaging (MRI) model integrating radiomics and DL techniques by utilizing ResNet-18 to accurately preoperatively stage laryngeal squamous cell carcinoma (LSCC) and predict progression-free survival, thereby improving clinical decision-making. Alazwari et al. (23) presented an efficient Laryngeal Cancer Detection using Chaotic Metaheuristics Integration with Deep Learning (LCD-CMDL) method integrating CLAHE for contrast enhancement, Squeeze-and-Excitation ResNet (SE-ResNet) for feature extraction, chaotic adaptive sparrow search algorithm (CSSA) for tuning, and extreme learning machine (ELM) for accurate classification. Dharani and Danesh (24) presented an improved DL ensemble method by incorporating enhanced EfficientNet-B5 with squeeze-and-excitation and hybrid spatial-channel attention modules and ResNet50v2, optimized by the tunicate swarm algorithm (TSA) for improving early and accurate diagnosis of oral cancer using the ORCHID histopathology image dataset. Majeed et al. (25) enhanced oral cavity squamous cell carcinoma (OCSCC) diagnosis using TL integrated with data-level imbalance handling techniques such as synthetic minority over-sampling technique (SMOTE), Deep SMOTE, ADASYN, and undersampling methods like Near Miss and Edited Nearest Neighbours to improve classification accuracy on imbalanced histopathological datasets. Song et al. (26) reviewed the role of AI in improving personalized management of head and neck squamous cell carcinoma (HNSCC) by integrating radiologic, pathologic, and molecular data for improved diagnosis, prognosis, treatment planning, and outcome prediction across the HNSCC care continuum. Kumar et al. (27) presented a Deep Learning Convolutional Neural Network (DL-CNN) model based on a modified Inception-ResNet-V2 architecture, using TL for the automated classification.

The existing studies exhibit various limitations, and a research gap exists in addressing diagnostic challenges under varied imaging conditions and lesion types. The NBI or WL are mainly utilized and fail to utilize multimodal data fusion effectively. Various techniques emphasize classification accuracy but lack interpretability, restricting clinical applicability. Moreover, optimization models such as WOA or TSA are not explored adequately in handling intrinsic tuning across hybrid DL techniques. Integration of spatial-channel attention and sequential learning remains inconsistent, while methods incorporating image and voice data are still in early experimentation stages. Hence, a significant research gap is in presenting robust, interpretable, and multimodal AI systems capable of real-time and accurate LC diagnosis across diverse clinical scenarios.

## 3 Methods

**Figure 1:**
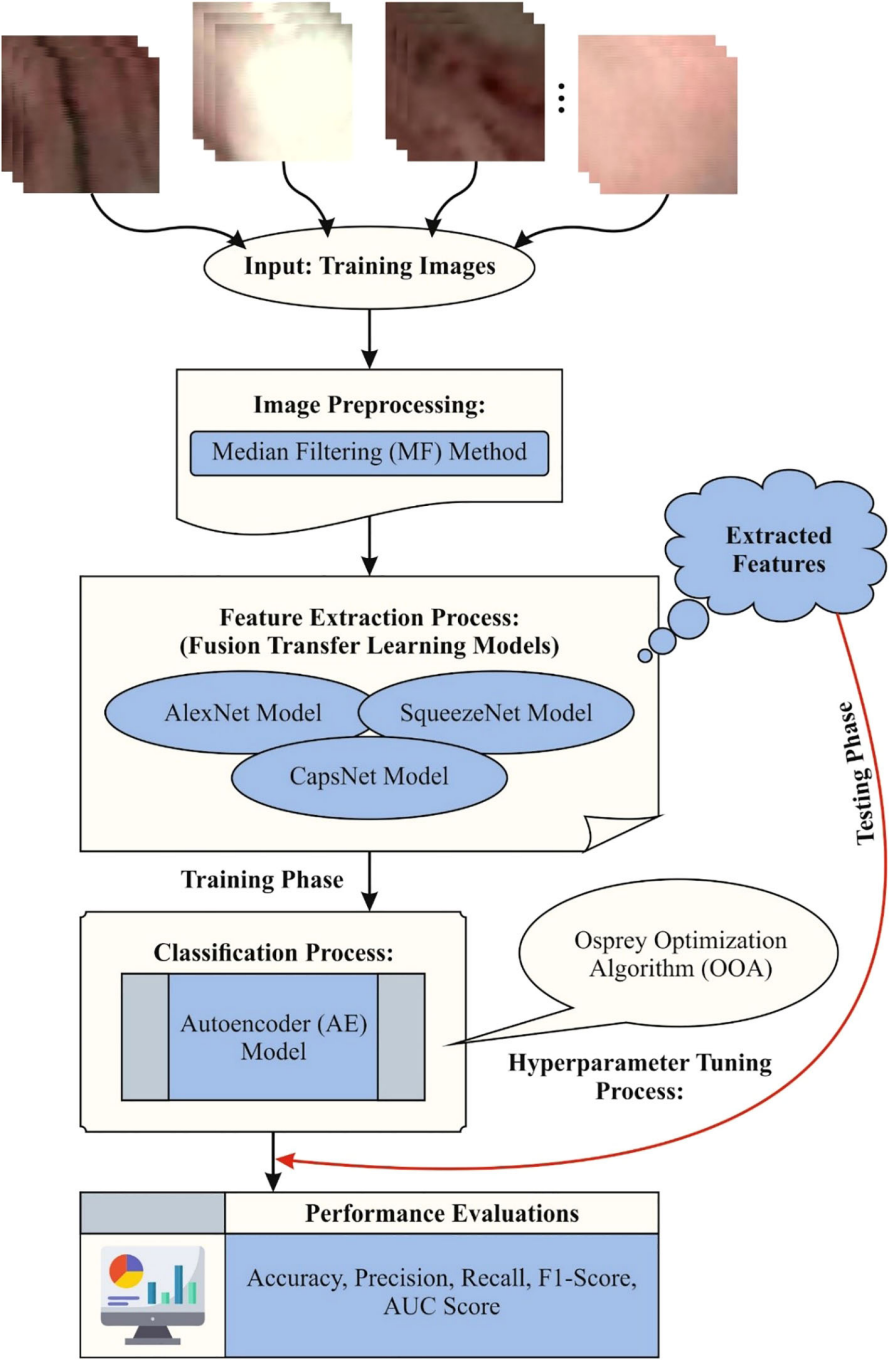
Workflow process of the CDLCHI-FTLOOA model.

The study proposed the CDLCHI-FTLOOA model for validation. The proposed method aims to improve the detection accuracy of LC using histology image analysis to increase the lifespan of the patients and their quality of life. The CDLCHI-FTLOOA model involves various steps such as the MF-based image pre-processing, feature extraction and classification, and parameter tuning. **Figure 1** illustrates the entire workflow process of the CDLCHI-FTLOOA technique.

### 3.1 MF-based image pre-processing

Initially, the CDLCHI-FTLOOA model utilizes the MF-based noise elimination for image pre-processing (28). This model is chosen for its efficiency in eliminating noise while conserving crucial edge details, making it appropriate for improving medical and microscopic images. Compared to other techniques like Gaussian or mean filtering, MF is more robust against salt-and-pepper noise and avoids blurring critical features. Furthermore, a 3×3 kernel size was used for this method, presenting a good balance between noise reduction and detail preservation. The application of MF resulted in noticeable enhancement in image quality, highlighting improved consistency and clarity. This pre-processing step contributed to better feature extraction and, ultimately, improved classification performance.

MF is a nonlinear image processing model applied to eliminate the noise while preserving the edges, making it an efficient option for histology image analysis. In terms of the LC detection process, it improves the quality of the image by smoothening the smaller devices without blurring critical cellular structures. This pre-processing stage helps achieve improved visualization and segmentation of the tissue features. MF is beneficial in removing the salt-and-pepper noise that influences the outcomes from histological slides. Enhancing image clarity helps in achieving precise classification and feature extraction. Finally, this step also improves the consistency of the automated diagnostic systems for LC.

### 3.2 Fusion of feature extraction methods

After image pre-processing, the feature extraction process is performed by the fusion models, namely, AlexNet, SqueezNet, and CapsNet. The AlexNet model provides robust basic features and is prevalent for its effective hierarchical feature learning and robustness in image recognition tasks. SqueezeNet presents a lightweight model with fewer parameters, ensuring faster processing and lower computational cost without losing accuracy, making it ideal for real-time applications. The spatial associations are effectively captured by the CapsNet model and also preserve pose feature information, which is significant for discriminating subtle differences in medical images like those of the larynx. Integrating these models allows for multiscale and diverse feature representation, enhancing discrimination between cancerous and non-cancerous regions. This hybrid approach overcomes limitations of individual networks and outperforms conventional single-model techniques in both accuracy and efficiency.

#### 3.2.1 AlexNet approach

AlexNet is a seminal structure in the domain of DL and is vital in transforming the tasks involved in image classification (29). AlexNet contains eight layers and presents an advanced procedure deploying five convolutional layers, injected with three fully connected (FC) layers. The input data of the method is designed in (64, 64, 3) sizes, representing 64 pixels in width and height, using three color channels. Then, the max-pooling layers using pool dimensions of 2×2 and a stride of 2 are combined. The 2^nd^ layer of the convolutional network includes 256 filters using kernel dimensions of 3×3 size and padding fixed to ‘same’, followed by other max pooling layers using the identical conditions as the previous layer. Each layer utilizes the ReLU activation function. The structure changes to the FC layer from the 6^th^ through the 8^th^ layers. The 6^th^ layer displays 4096 neurons, all using the ReLU activation functions, accompanied by a dropout layer using a standardization rate of 0.5. The 7^th^ layer imitates the 6^th^ framework. During the 8^th^ layer, the number of neurons is decreased to 5 using the Softmax activation function to enable multi-class classification. The method is created using the Adam optimizer. Categorical cross-entropy acts as the loss function, whereas accuracy is accepted as the evaluation method.

#### 3.2.2 SqueezNet method

**Figure 2:**
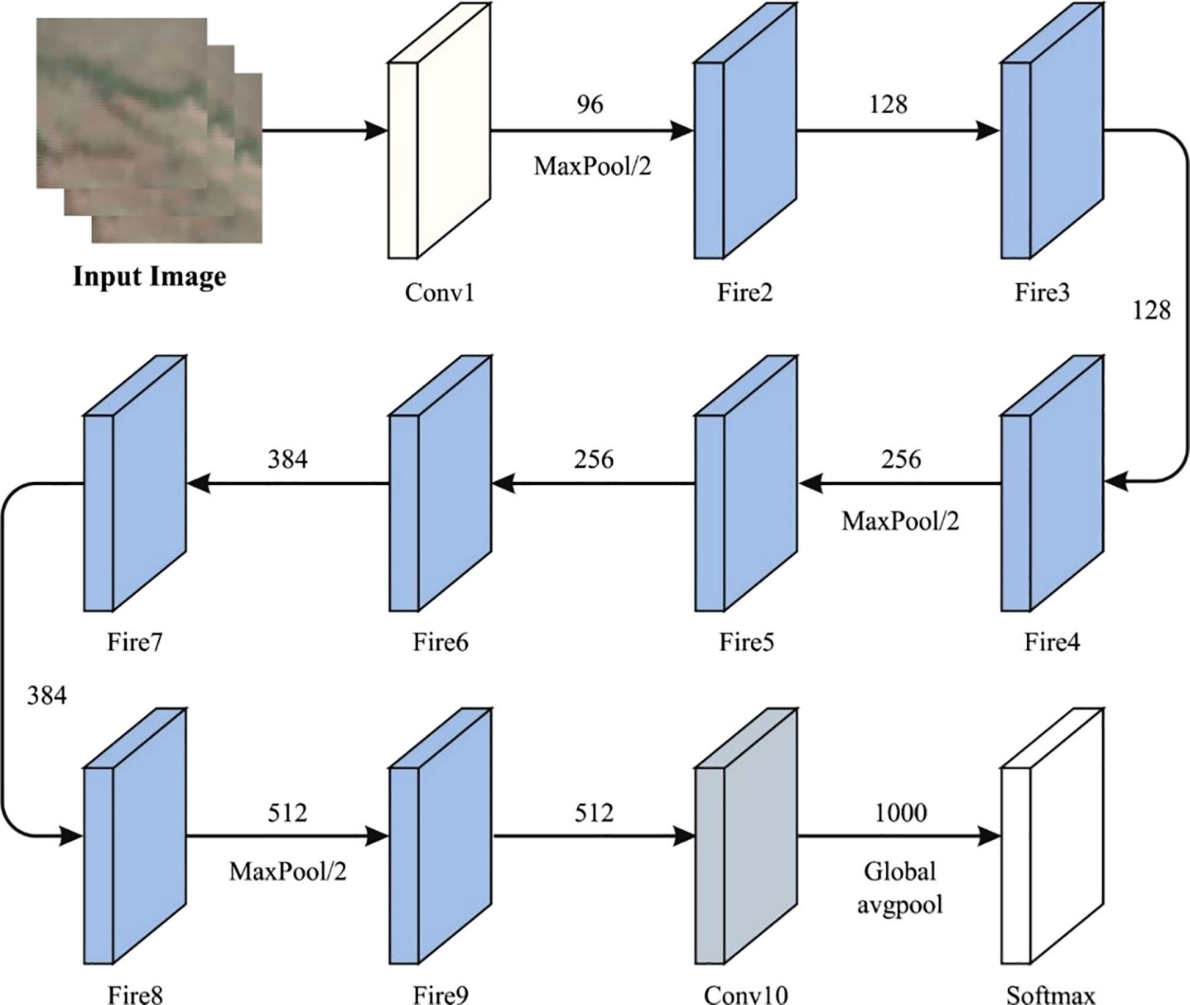
Architecture of the SqueezNet technique.

SqueezeNet is a special DCNN structure that is specially designed for effective and low‐power outcome (30). It is tailored to attain higher precision in image classification tasks while reducing computational resources and the model’s size. The structure begins with an input tensor (64, 64, 3) and three color components (RGB). Then, it upgrades the over-layer sequences that contain pooling and convolutional processes. Particularly, the SqueezeNet method combines the distinguishing features named ‘fire modules’. This module includes parallel 1×1 and 3×3 convolutions that are tailored for balancing both model clarity and computational complexity. Following the fire units, the system incorporates additional convolutional layers, a dropout layer for normalization, a 1×1 convolution layer to improve the attributes, and a global average pooling layer for reducing the dimensions. This structure results in a dense layer output using the Softmax activation function, enabling multi-class classification. Categorical cross-entropy is applied as the loss function. The performance of the method is assessed according to accuracy, a metric used to determine its efficiency in the precise categorization of the images. It outshines its capability to attain higher accuracy in image classification tasks, thus making it very important for settings where effectiveness and lower‐power conclusions are dominant. **Figure 2** shows the architecture of the SqueezNet technique.

#### 3.2.3 CapsNet model

CapsNet is an advanced technology in DL methods and is specifically suitable for challenges and advantages from the hierarchical framework of information (31). Here, the capsule is a kind of neuron whose outputs signify diverse assets of similar entities. The dissimilar neurons are present in the CNN scalar output and the output vector of the capsules. At the same time, the positioning of the assets and the features represents the likelihood of a feature. The fundamental structure contains basic and digit capsules, succeeded by dynamic routing mechanisms. The significant parameter for the CapsNet structure remains the squashing function, *S*.


(1) $$S\;=\frac{\|v_{j}{\|}^{2}}{1+\|v_{j}{\|}^{2}}\frac{v_{j}}{\left\|v_{j}\right\|}$$

In Equation 1, ‖·‖ is the Euclidean norm function and $v_{j}$ signifies the output vector of the capsule j . S guarantees the output vector length between 0 and 1. The squashing function ensures that the length of all the capsule’s vector output lies between 0 and 1. Here, the length signifies the likelihood of the existence of the features. This function upholds the orientation of the vector encoding significant data about the recognized features, like scale or rotation.

The central capsule is a primary layer of capsules that carries out the primary higher‐dimension entity recognition in the images. The initial capsules produce the basic layer, which promptly connects with the raw features and is then removed by the primary convolution layer. These initial capsules acquire the scalar output from the convolution layer and modify it as the output vector, which in turn depicts the instantiating parameter of several aspects. Numerous primary capsules are usually selected based on the intricacy of the features in the database. In the case of simple databases, some primary capsules are adequate. However, complex databases with diverse spatial hierarchies can necessitate a considerable volume of capsules to acquire the relations sufficiently. The selection of the initial capsules is frequently associated with analytical outcomes and directed by the execution of the system using the benchmark data. It acquires the feature mapping process formed by classical convolution layers and modifies them into smaller vectors that transfer precise data. The vector of prediction $U_{ij}$ by capsule *i* for capsule *j* is calculated as shown below.


(2) $$U_{ij}=W_{ij}u_{i}$$

In Equation 2, $u_{i}$ is the output of capsule *j*, and $W_{ij}$ specifies the weighted matrix between capsules *i* and *j*. This equation defines the capsules in a single layer, which is then communicated to the subsequent layer. The weighted matrix $W_{ij}$ mainly establishes the transition of the data and its exchange among the layers. It makes the prediction $U_{ij}$ as input and employs the routing process through agreement mechanisms for generating the last output.


(3) $$c_{ij}=\frac{\exp(b_{ij})}{\sum_{k}\text{exp}(b_{ik})}$$

In Equation 3, $b_{ij}$ refers to the initial login possibilities in which the capsule *i* must be together with capsule *j*, and *k* represents the probable parent capsule counts in the above layer. This procedure guarantees that the capsules with aligned outputs increase their connection. The complete input of $s_{j}$ denotes the sum of weighted prediction vectors as shown in Equation 4:


(4) $$s_{ij}=\sum_{k}c_{ij}U_{ij}$$

The output of the *j* ^th^ capsule has only the complete squashed input as given below.


(5) $$v_{j}=S(s_{j})$$

After adding the inputs from low‐level capsules, the system integrates the data collected from diverse segments of the image, thus allowing the high‐level capsules to make additionally accurate and complex feature models. Equation 5 refers to the vector whose length signifies the existing features, whereas its orientation encodes the additional assets, thus preserving the crucial spatial particulars of the feature. The last phase of this method is the dynamical routing process.

### 3.3 AE-based classification process

In general, the AE model is employed for the classification process (32). This model is chosen for its robust capability in unsupervised feature learning, which effectively compresses high-dimensional data into a lower-dimensional representation while preserving essential data. The redundant features and noise are mitigated by this model, thus improving the generalization and robustness. Unlike conventional classifiers, the AE model efficiently learn complex data patterns through reconstruction, which also enhances classification accuracy, specifically in intrinsic medical images. Moreover, the ability of AE to perform dimensionality reduction minimizes computational costs and overfitting risks compared to standard deep networks. This makes AE-based classification particularly appropriate for handling the rich and complex features extracted from fusion CNN models in LC detection.

AE is a form of unsupervised DL method that is mainly used for feature extraction and dimensionality reduction processes. The basic concept behind the AEs is to learn a compressed format (encoder) of the input data, after which a new input is built based on the condensed representations. The structure of the AE contains dual basic elements, such as a decoder and an encoder. The encoding process condenses the input, whereas the decoding process recreates the input from the encoded information. The AEs are trained to reduce the change between the input and its rebuilt form, utilizing the loss function to calculate the reconstruction error. The structure is explained as follows.

Encoder: The encoder condenses the input data into a low‐dimensional space by removing the crucial attributes. It maps the input data XX to the latent area representation, denoted by Z .


(6) $$Z=f(X)=\sigma (W_{e}X+b_{e})$$

In Equation 6, $W_{e}\;$ characterize the weights, $b_{e}$ denotes the biases and σ refers to the activation function, such as ReLU or sigmoid.

Latent Space: The compressed or the encoded representation, i.e., ZZ, represents the bottleneck in the system. This low-dimensional representation makes the AEs an efficient candidate for detecting the anomalies, as it removes the noise and unrelated characteristics.

Decoder: The decoding process rebuilds the new information from the condensed latent area. It maps the ZZ and reverts to the input area, thus making a reconstruction $X^{\wedge }X^{\wedge }$ :


(7) $$\hat{X}=g(Z)=\sigma (W_{d}Z+b_{d})$$

In Equation 7, $b_{d}$ and $W_{d}$ characterize the biases and weights of the decoder, correspondingly. The reconstruction error that estimates the change between the new input XX and its reconstruction $X^{\wedge }X^{\wedge }$ , is reduced during the training process. The aim is to learn the condensed representation to retain the most significant data, see Equation 8.


(8) $$L(X,\hat{X})=\frac{1}{n}\sum_{i=1}^{n}{\left(X_{i}-\hat{X}\right)}^{2}$$

The learning procedure of the AEs includes the optimization of the biases and weights to reduce the reconstruction error. The training procedure is outlined in the succeeding phases.

Encoder Stage: The encoding condenses the input data into latent area representations as given below in Equation 9.


(9) $$Z=\sigma (W_{e}X+b_{e})$$

Decoder Stage: The decoder rebuilds the data from the latent representations as given below in Equation 10.


(10) $$\hat{X}=\sigma (W_{d}Z+b_{d})$$

Loss Function: The reconstruction error is reduced during the training as given below in Equation 11.


(11) $$L=\frac{1}{n}\sum_{i=1}^{n}{\left(X_{i}-\hat{X}\right)}^{2}$$

Here, $X_{i}$ embodies the new input, and $\hat{X}$ refers to the reconstructed output. Meanwhile, the method is enhanced to rebuild the typical formats, while some critical changes occur in the reconstruction error. By taking the inherent architecture of the standard designs, the AEs may detect subtle anomalies often overlooked by other methods. Finally, the AEs function as a powerful and efficient approach to detecting cancer. The ability of the AEs to reduce the dimensions while preserving the essential features allows them to distinguish between standard and doubtful actions, thus making them a beneficial device in the cybersecurity area.

### 3.4 Parameter tuning using the OOA model

To further optimize the performance of the model, the OOA model is utilized for hyperparameter tuning to select the best hyperparameters for enhanced accuracy (33). This model is chosen for its biologically inspired search mechanism, which also effectually balances exploration and exploitation to avoid local optima. The model also shows limitations in conventional methods and improves convergence speed and accuracy when fine-tuning hyperparameters, resulting in an enhanced model performance. Compared to other metaheuristic algorithms, OOA illustrate robustness in handling complex, high-dimensional search spaces, making it appropriate for optimizing DL methods such as the AE classifier. Its efficiency in finding optimal solutions mitigates training time and computational cost, thereby improving the overall efficiency of the LC detection framework.

OOA is a bio‐inspired metaheuristic model that emulates the Osprey’s approach in searching and carrying fish to an appropriate position for consumption. Primarily, the intellectual behavior of the Ospreys is mathematically expressed for resolving the optimization issues over three phases, such as the exploitation, exploration, and initialization, as briefed below.

#### 3.4.1 Initialization phase

During OOA, all the Ospreys depict population members that establish the solutions to the issue depending on their location in the searching area. A single osprey jointly makes the OOA population described by the matrix of dimension (N Osprey * X Position) that is initialized arbitrarily in the searching area, as shown in Equation 12.


(12) $$\begin{array}{c} \begin{array}{c} OS\\ Osprey\\ Population\;matrix \end{array}=[\begin{array}{l} OS_{1}\\ \vdots \\ OS_{i}\\ \vdots \\ OS_{n} \end{array}]\\ ={\left[\begin{array}{l} OS_{1,1}\\ \vdots \\ OS_{i,1}\\ \vdots \\ OS_{n,1} \end{array}\begin{array}{cccc} \cdots & OS_{1,j} & \cdots & OS_{1,m}\\ \ddots & \vdots & \ddots & \vdots \\ \cdots & OS_{i,j} & \cdots & OS_{i,m}\\ \ddots & \vdots & \ddots & \vdots \\ \cdots & OS_{n,j} & \cdots & OS_{n,m} \end{array}\right]}_{\begin{array}{c} n\\ Number\\ of\;OS \end{array}\;\;\times \begin{array}{c} m\\ Number\;of\\ problem\;\mathrm{var}iables \end{array}} \end{array}$$

In Equation 13, *OS_i_* represents the *i^th^* location of the OS.


(13) $$\begin{array}{c} \begin{array}{c} OS_{i,j}\\ Osprey\;i^{th}\;position\\ and\;j^{th}\;dimension \end{array}\\ =LB_{j}+rand_{i,j}[0,1].(\begin{array}{c} UB_{j}\\ Upper\;Bound\\ of\;j^{th}\;dimension \end{array}\;\;-\;\;\begin{array}{c} LB_{j}\\ Lower\;Bound\\ of\;j^{th}\;dimension \end{array}) \end{array}$$

Every OS in the OOA depicts a candidate solution for the optimization problem; thus, a fitness evaluation should be accomplished for every OS, as shown in Equation 14.


(14) $$\begin{array}{c} FF\\ Fitness\\ Function \end{array}={\left[\begin{array}{l} FF_{1}\\ \vdots \\ FF_{i}\\ \vdots \\ FF_{n} \end{array}\right]}_{n\times 1}={\left[\begin{array}{l} FF{\left(OS\right)}_{1}\\ \vdots \\ FF{\left(OS\right)}_{i}\\ \vdots \\ FF{\left(OS\right)}_{n} \end{array}\right]}_{n\times 1}$$

The position of the OSs is upgraded after assessing the worst, sub-optimal, or best values based on the fitness function (FF).

##### 3.4.1.1 Exploration stage

Ospreys have sharp eyesight that helps them locate underwater prey. It initiates the attack by segmenting the water to capture its target. Likewise, the real behavior of the OS threat on fish in searching areas can be mimicked by upgrading the location of the OS population, as it can enhance the exploration approach of OOA. Further, it also helps in establishing the finest position by avoiding local solutions. The location of the OS in the searching space holds a superior FF value, equivalent to the individual OS in OOA, as shown in Equation 15.


(15) $$\begin{array}{c} \underset{\begin{array}{c} Set\;of\;fish\;position\\ of\;i^{th}\;OS \end{array}}{\underset{︸}{Fposition_{i}}}=\{OS_{k}|k\in \{1,2,\ldots ,n\}\wedge FF_{k}<FF_{i}\}\\ \cup \{\begin{array}{l} OS_{best}\\ OS\;best\;position \end{array}\} \end{array}$$

The OS searches for its prey or underwater fish by arbitrarily establishing its positions, as shown in Equations 16 and 17.


(16) $$\begin{array}{c} \underset{\begin{array}{c} Updated\;position\;of\;i^{th}\\ OS\;j^{th}\;dimension\;in\;OOA \end{array}}{\underset{︸}{OS_{i,j}^{pos1}}}\\ =OS_{i,j}+rand_{i,j}[0,1]\cdot (\begin{array}{c} CF_{i,j}\\ Chosen\;fish\;of\;i^{th}\\ OS\;j^{th}\;dimension \end{array}\;\;-rand_{i,j}[1,2]\cdot oS_{i,j}) \end{array}$$

(17) $$OS_{i,j}^{pos1}=\{\begin{array}{l} OS_{i,j}^{pos1},LB_{j}\leq OS_{i,j}^{pos1}\leq UB_{j};\\ LB_{j},OS_{i,j}^{pos1}<LB_{j};\\ UB_{j},OS_{i,j}^{pos1}>UB_{j}; \end{array}$$

The novel value gets enhanced by utilizing the FF value in Equation 18.


(18) $$OS_{i}=\{\begin{array}{l} OS_{i}^{pos1},FF_{i}^{pos1}<FF_{i};\\ OS_{i},else \end{array}$$

#### 3.4.2 Exploitation phase

After searching, the OS decides the best location for eating the prey. The position of the OS in the searching space is modified as it holds the prey in a safe and suitable place. This approach enhances the local searching exploitation capability and converges to the finest solution. In this design stage of the OOA, Equation 19 specifies the behavior of the model and establishes its arbitrary selection location to consume the prey.


(19) $$\begin{array}{c} \underset{\begin{array}{c} New\;position\;of\;i^{th}\\ OS\;j^{th}\;dimension\;2^{nd}\;phase\;of\;OOA \end{array}}{\underset{︸}{OS_{i,j}^{pos2}}}\\ =OS_{i,j}+\frac{LB_{j}+rand_{i,j}[0,1]\cdot (UB_{j}-LB_{j})}{k} \end{array}$$

(20) $$OS_{i,j}^{pos2}=\{\begin{array}{l} OS_{i,j}^{pos2},LB_{j}\leq OS_{ij}^{pos2}\leq UB_{j};\\ LB_{j},OS_{i,j}^{pos2}<LB_{j};\\ UB_{j},OS_{i,j}^{pos2}>UB_{j}; \end{array}$$

Here, Equation 20 enhances the calculated value of the FF after the previous position of the OS is updated. Fitness selection is a significant feature that prompts the performance of the OOA. The hyperparameter selection model consists of a solution encoding model to calculate the effectiveness of the candidate solutions. In this study, the OOA imitates ‘accuracy’ as the leading standard to model the FF, as shown below, as shown in Equations 21 and 22.


(21) Fitness = max (P)

(22) $$P=\frac{TP}{TP+FP}$$

Here, TP and FP depict the true and false positive rates, respectively.

## 4 Experimental outcomes

**Figure 3:**
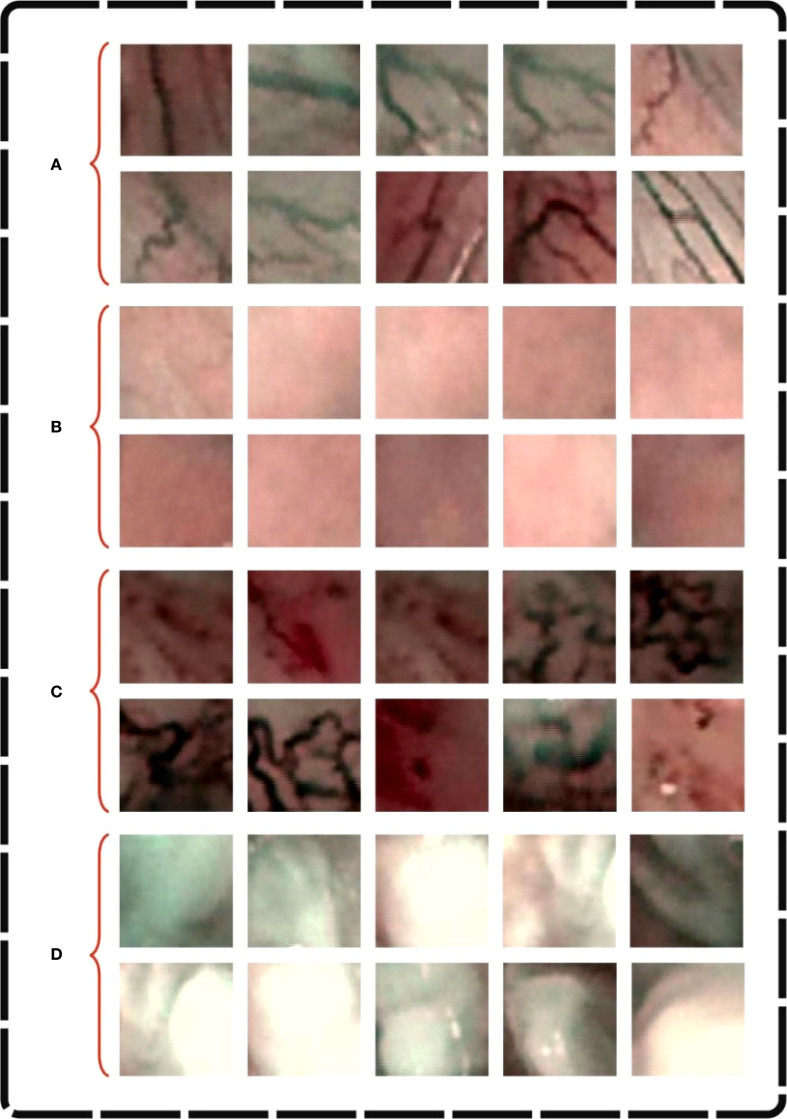
Sample images of **(A)** Hbv, **(B)** He, **(C)** IPCL and **(D)** Le.

This section discusses the experimental outcomes of the CDLCHI-FTLOOA model under the laryngeal dataset (34). This dataset comprises 1,320 patches of early-stage and healthy cancerous laryngeal tissues, classified under four classes, namely, Hypertrophic Blood Vessels (HBV), Healthy Tissue (He), Abnormal IPCL-like Vessel (IPCL), and Leukoplakia (Le). The patches (100x100 pixels) were extracted from 33 narrow-band laryngoscopic images of 33 dissimilar patients, affected by laryngeal spinocellular carcinoma (analyzed after histopathological inspection). The complete details of this dataset are shown in **Table 1**. **Figure 3** illustrates a set of sample images.

**Table 1:** Details of the dataset.

Tissue classes	No. of images
Hbv	330
He	330
IPCL	330
Le	330
Total Images	1320

**Figure 4:**
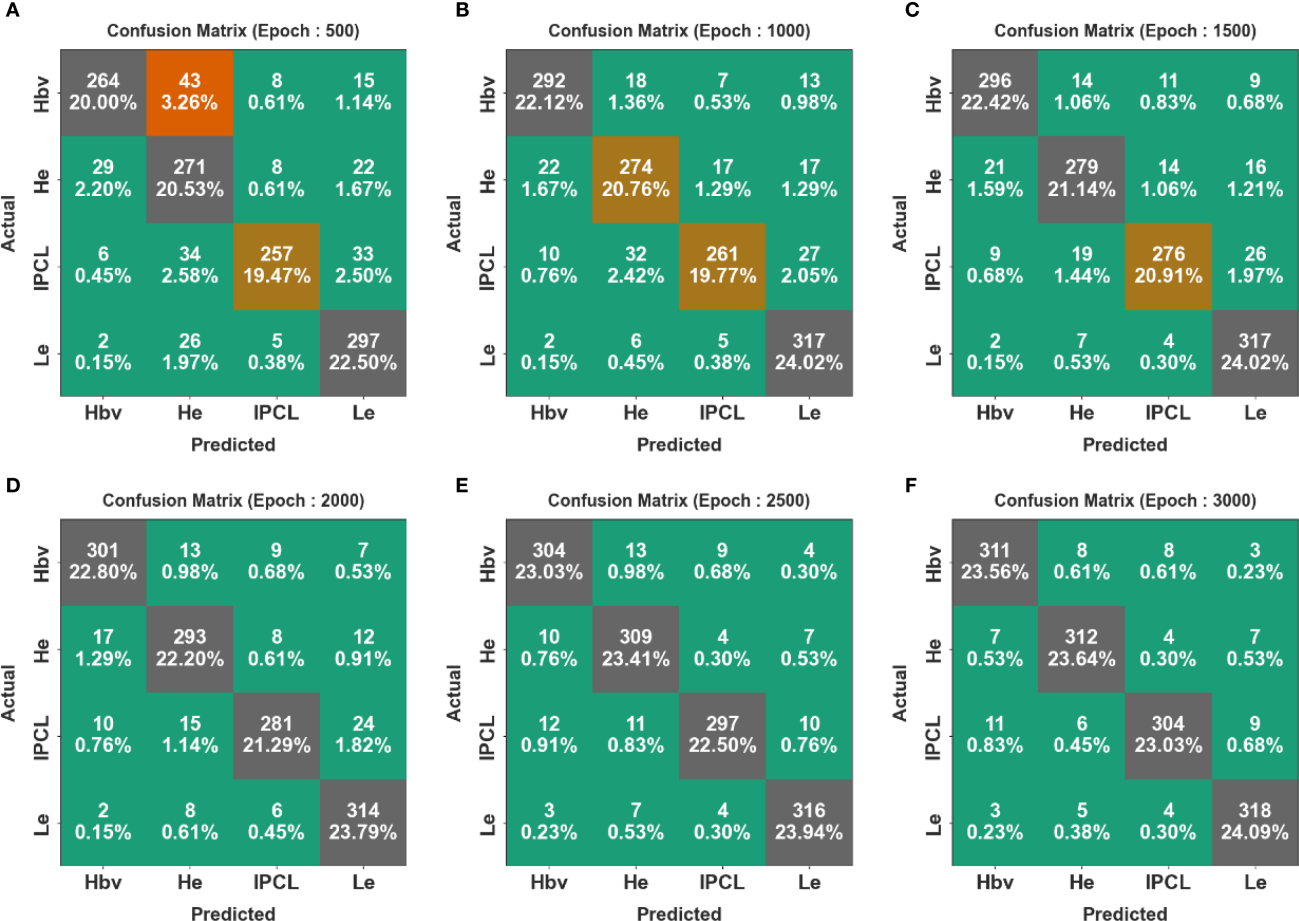
Confusion matrices of the CDLCHI-FTLOOA model **(A–F)**, Epochs 500-3000.

**Figure 4** shows the confusion matrices generated by the CDLCHI-FTLOOA method under a diverse number of epochs. The results infer that the CDLCHI-FTLOOA method successfully identified and detected all four classes accurately. The LC detection result of the CDLCHI-FTLOOA approach is defined in terms of different numbers of epochs in **Table 2**.

**Table 2:** LC detection outcomes of the CDLCHI-FTLOOA model under a distinct number of epochs.

Class labels	$Accu_{y}$	$Prec_{n}$	$Reca_{l}$	$F1_{score}$	$AUC_{score}$
Epoch - 500					
Hbv	92.20	87.71	80.00	83.68	88.13
He	87.73	72.46	82.12	76.99	85.86
IPCL	92.88	92.45	77.88	84.54	87.88
Le	92.20	80.93	90.00	85.22	91.46
Average	91.25	83.39	82.50	82.61	88.33
Epoch - 1000					
Hbv	94.55	89.57	88.48	89.02	92.53
He	91.52	83.03	83.03	83.03	88.69
IPCL	92.58	90.00	79.09	84.19	88.08
Le	94.70	84.76	96.06	90.06	95.15
Average	93.33	86.84	86.67	86.58	91.11
Epoch - 1500					
Hbv	95.00	90.24	89.70	89.97	93.23
He	93.11	87.46	84.55	85.98	90.25
IPCL	93.71	90.49	83.64	86.93	90.35
Le	95.15	86.14	96.06	90.83	95.45
Average	94.24	88.58	88.48	88.43	92.32
Epoch - 2000					
Hbv	95.61	91.21	91.21	91.21	94.14
He	94.47	89.06	88.79	88.92	92.58
IPCL	94.55	92.43	85.15	88.64	91.41
Le	95.53	87.96	95.15	91.41	95.40
Average	95.04	90.16	90.08	90.05	93.38
Epoch - 2500					
Hbv	96.14	92.40	92.12	92.26	94.80
He	96.06	90.88	93.64	92.24	95.25
IPCL	96.21	94.59	90.00	92.24	94.14
Le	97.35	93.77	95.76	94.75	96.82
Average	96.44	92.91	92.88	92.87	95.25
Epoch - 3000					
Hbv	96.97	93.67	94.24	93.96	96.06
He	97.20	94.26	94.55	94.40	96.31
IPCL	96.82	95.00	92.12	93.54	95.25
Le	97.65	94.36	96.36	95.35	97.22
Average	97.16	94.32	94.32	94.31	96.21

**Figure 5:**
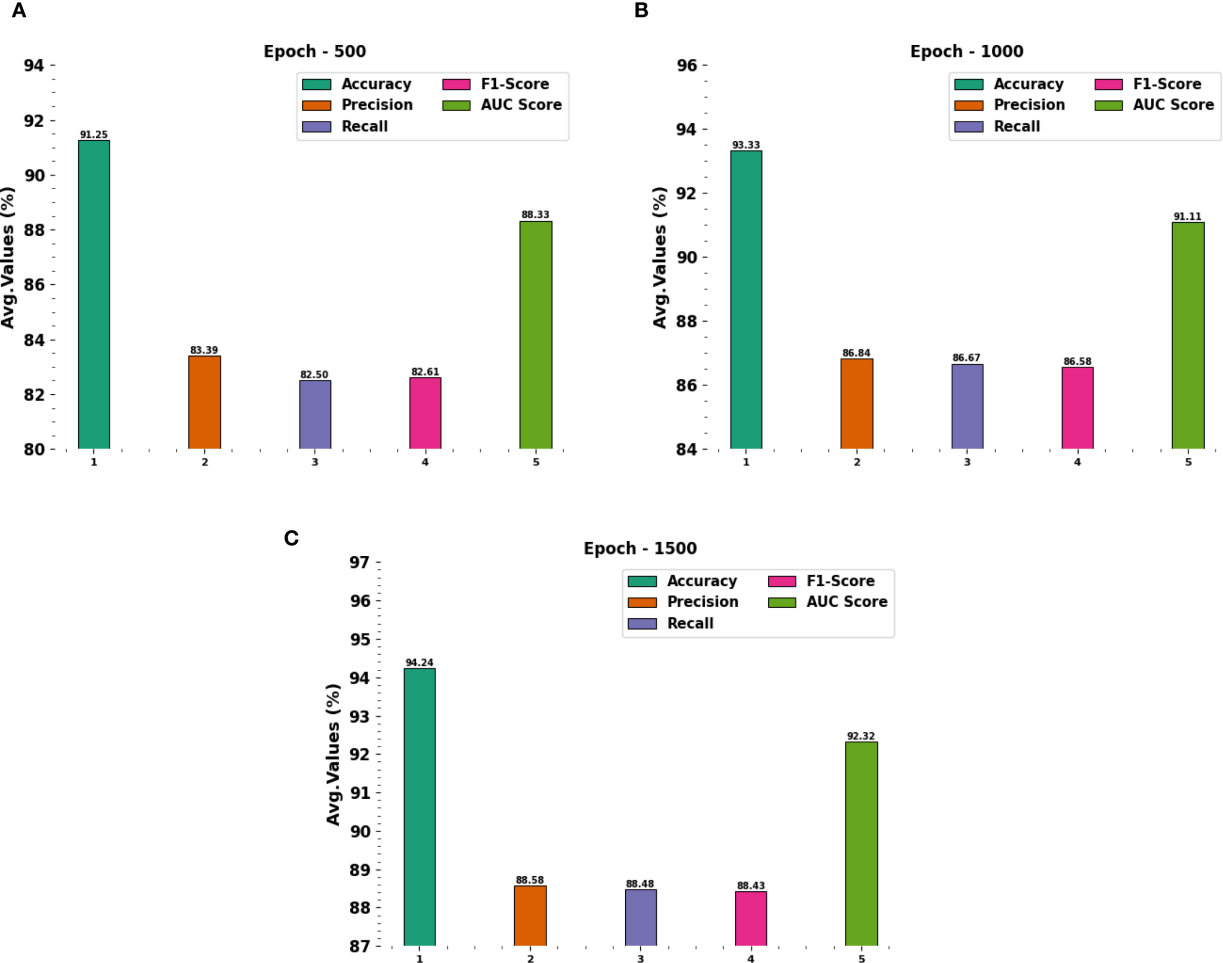
Average outcomes of the CDLCHI-FTLOOA model **(A–C)**, Epochs 500-1500.

**Figure 5** portrays the average outcomes achieved by the CDLCHI-FTLOOA technique under 500 to 1500 epochs. On 500 epochs, the CDLCHI-FTLOOA technique achieved the average $accu_{y}$ , $prec_{n}$ , $reca_{l}$ , $F1_{score}$ , and $AUC_{score}$ of 91.25%, 83.39%, 82.50%, 82.61%, and 88.33% respectively. Moreover, on 1,000 epochs, the CDLCHI-FTLOOA technique attained an average $accu_{y}$ , $prec_{n}$ , $reca_{l}$ , $F1_{score}$ , and $AUC_{score}$ of 93.33%, 86.84%, 86.67%, 86.58%, 91.11% correspondingly. Additionally, on 1,500 epochs, the CDLCHI-FTLOOA method achieved an average $accu_{y}$ , $prec_{n}$ , $reca_{l}$ , $F1_{score}$ , and $AUC_{score}$ of 94.24%, 88.58%, 88.48%, 88.43%, 92.32% respectively.

**Figure 6:**
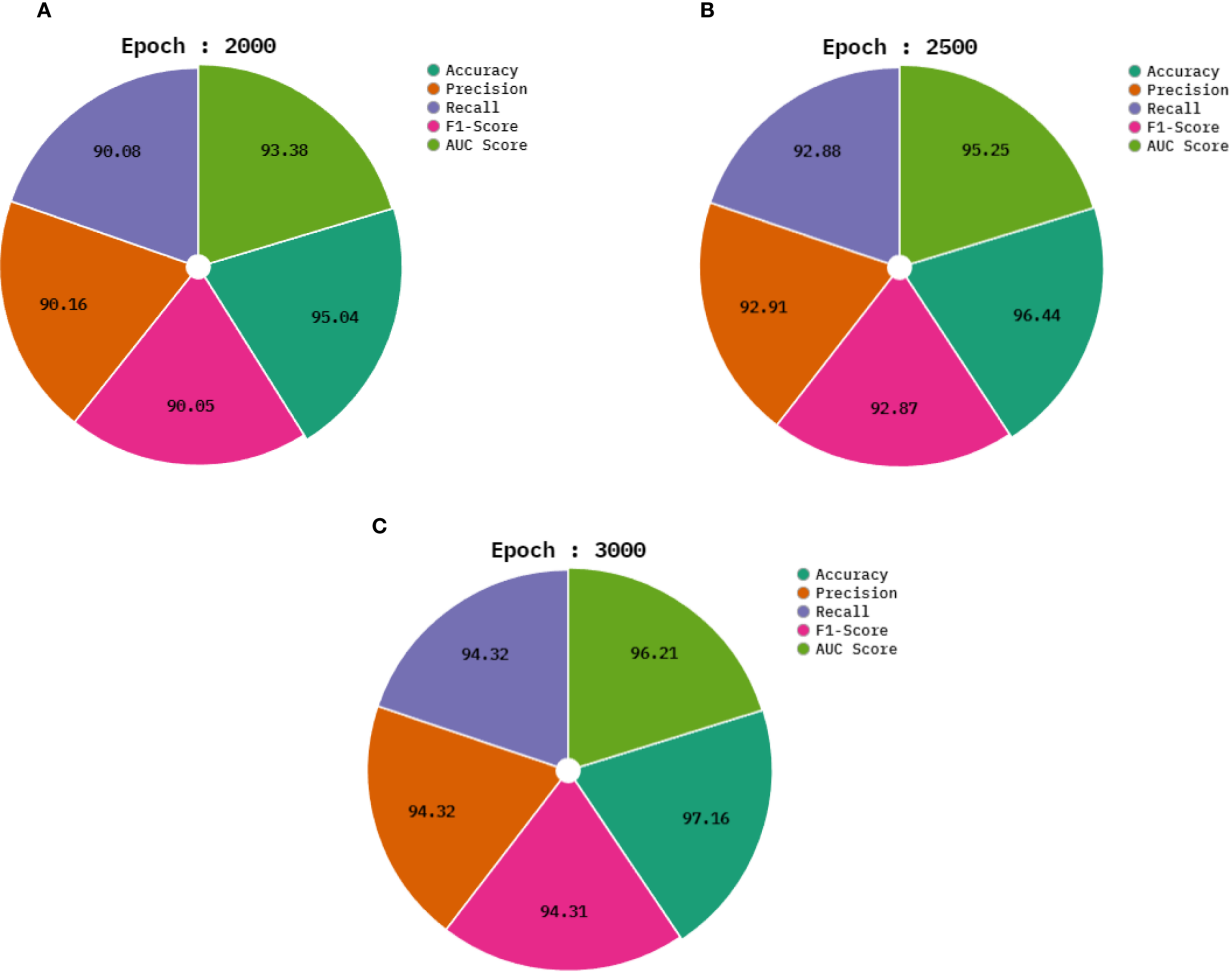
Average outcomes of the CDLCHI-FTLOOA model **(A–C)**, Epochs 2000-3000.

In **Table 2** and **Figure 6**, the average outcomes of the CDLCHI-FTLOOA technique under 2000 to 3000 epochs are shown. On 2,000 epochs, the CDLCHI-FTLOOA model attained an average $accu_{y}$ , $prec_{n}$ , $reca_{l}$ , $F1_{score}$ , and $AUC_{score}$ of 95.04%, 90.16%, 90.08%, 90.05%, 93.38% respectively. In addition to this, on 2,500 epochs, the CDLCHI-FTLOOAs model attained an average $accu_{y}$ , $prec_{n}$ , $reca_{l}$ , $F1_{score}$ , and $AUC_{score}$ of 96.44%, 92.91%, 92.88%, 92.87%, 95.25% correspondingly. Furthermore, on 3,000 epochs, the CDLCHI-FTLOOAs model attained an average $accu_{y}$ , $prec_{n}$ , $reca_{l}$ , $F1_{score}$ , and $AUC_{score}$ of 97.16%, 94.32%, 94.32%, 94.31%, 96.21%, respectively.

**Figure 7:**
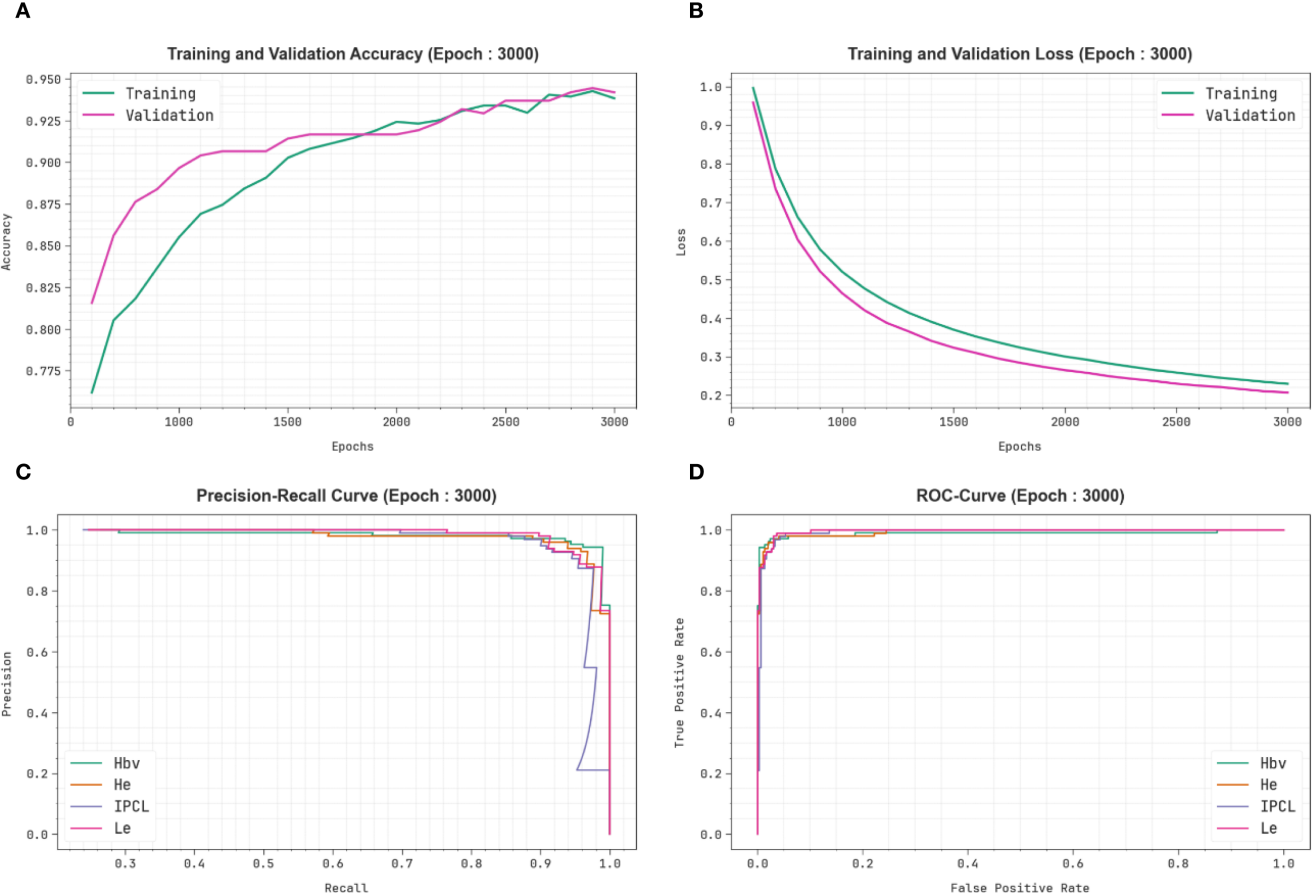
CDLCHI-FTLOOA technique of **(A)** Accuracy, **(B)** Loss, **(C)** PR curve, and **(D)** ROC curve.

**Figure 7** exhibits the classification outcomes of the CDLCHI-FTLOOA model. **Figure 7A** depicts the accuracy analysis results attained by the CDLCHI-FTLOOA model. The figure infers that the CDLCHI-FTLOOA method achieved increasing values over an increasing number of epochs. Next, **Figure 7B** exemplifies the result from the loss analysis of the CDLCHI-FTLOOA method. The outcomes specify that the proposed method accomplished closer training and validation loss values. **Figure 7C** reveals the PR examination outcome of the CDLCHI-FTLOOA technique. The findings indicate that the CDLCHI-FTLOOA technique produced an increase in the PR values. Lastly, **Figure 7D** portrays the ROC analysis outcome of the CDLCHI-FTLOOA technique. The figure infers that the projected system increased the ROC values.

**Figure 8:**
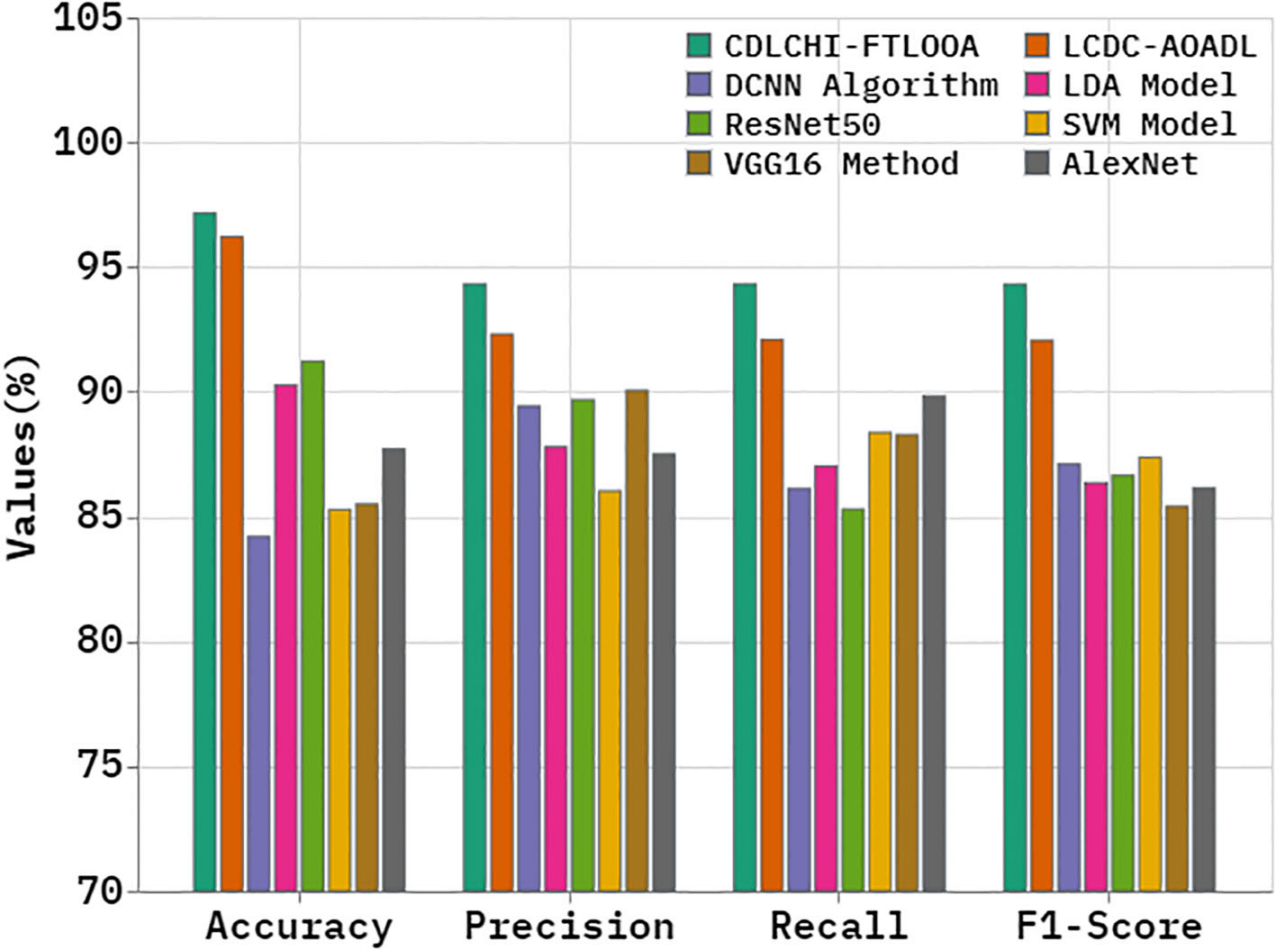
Comparative analysis outcomes of the CDLCHI-FTLOOA model with existing methodologies.

A comparative analysis was conducted between the CDLCHI-FTLOOA method and other advanced techniques, and the outcomes are shown in **Table 3** and **Figure 8** (5, 35). In terms of $accu_{y}$ , the CDLCHI-FTLOOA method achieved a maximum $accu_{y}$ of 97.16%. In contrast, the LCDC-AOADL, DCNN, LDA, ResNet50, SVM, VGG16, and AlexNet models attained lower $accu_{y}$ values such as 96.21%, 84.20%, 90.27%, 91.22%, 85.28%, 85.50%, and 87.71%, correspondingly. Similarly, in terms of $prec_{n}$ , the CDLCHI-FTLOOA method accomplished a maximum $prec_{n}$ of 94.32% while the LCDC-AOADL, DCNN, LDA, ResNet50, SVM, VGG16, and AlexNet models achieved the least $prec_{n}$ values, such as 92.31%, 89.43%, 87.79%, 89.68%, 86.02%, 90.05%, and 87.51%, respectively. In terms of $F1_{score}$ , the CDLCHI-FTLOOA technique attained the highest $F1_{score}$ of 94.31% while the LCDC-AOADL, DCNN, LDA, ResNet50, SVM, VGG16, and AlexNet models attained lower $F1_{score}$ values, such as 92.06%, 87.10%, 86.35%, 86.65%, 87.37%, 85.41%, and 86.15%, correspondingly.

**Table 3:** Comparative analysis outcomes of the CDLCHI-FTLOOA model with existing methodologies.

Methodology	$Accu_{y}$	$Prec_{n}$	$Reca_{l}$	$F1_{score}$
CDLCHI-FTLOOA	97.16	94.32	94.32	94.31
LCDC-AOADL	96.21	92.31	92.09	92.06
DCNN Algorithm	84.20	89.43	86.12	87.10
LDA Model	90.27	87.79	87.02	86.35
ResNet50	91.22	89.68	85.30	86.65
SVM Model	85.28	86.02	88.37	87.37
VGG16 Method	85.50	90.05	88.28	85.41
AlexNet	87.71	87.51	89.83	86.15

**Figure 9:**
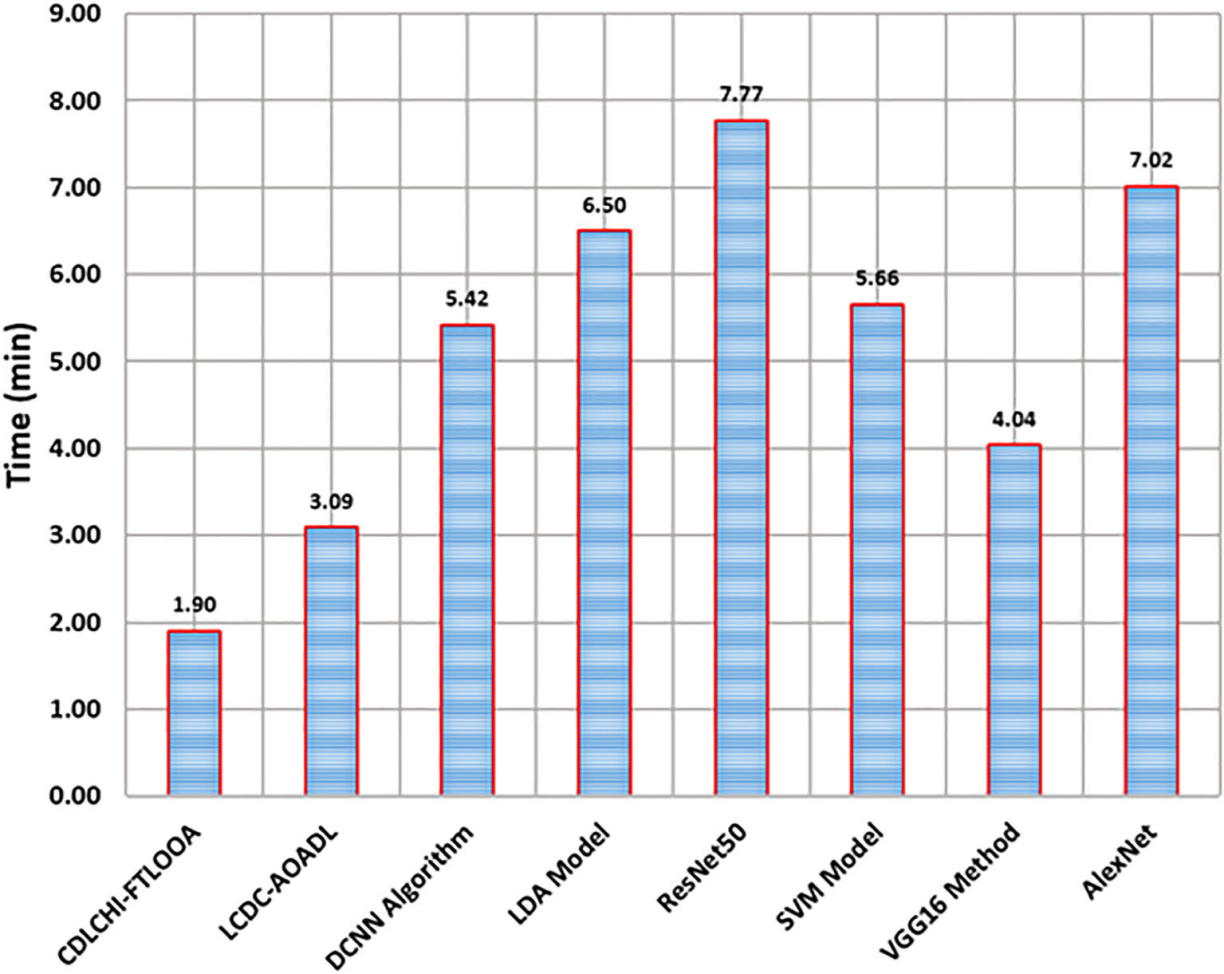
Time outcomes of the CDLCHI-FTLOOA technique with existing methods.

**Table 4** and **Figure 9** demonstrate the comparative analysis outcomes of the CDLCHI-FTLOOA method and other methods in terms of execution time. The introduced CDLCHI-FTLOOA technique consumed the least possible time, i.e., 1.90min, whereas the LCDC-AOADL, DCNN, LDA, ResNet50, SVM, VGG16, and AlexNet methods took larger times, such as 3.09min, 5.42min, 6.50min, 7.77min, 5.66min, 4.04min, and 7.02min, respectively.

**Table 4:** Time outcome of the CDLCHI-FTLOOA technique with existing methods.

Methodology	Time (min)
CDLCHI-FTLOOA	1.90
LCDC-AOADL	3.09
DCNN Algorithm	5.42
LDA Model	6.50
ResNet50	7.77
SVM Model	5.66
VGG16 Method	4.04
AlexNet	7.02

## 5 Conclusion

In this study, the CDLCHI-FTLOOA model was proposed. The model aimed to improve the detection accuracy of LC using histology image analysis to improve patient outcomes. Initially, the CDLCHI-FTLOOA model utilized MF-based noise elimination during the image pre-processing stage. Furthermore, the feature extraction process was conducted using fusion models, namely AlexNet, SqueezNet, and CapsNet. The AE model, used for classification, was further optimized using OOA for hyperparameter tuning to enhance accuracy by choosing the best parameters. To exhibit the improved performance of the CDLCHI-FTLOOA model, a comprehensive experimental analysis was conducted under the laryngeal dataset. The comparison study of the CDLCHI-FTLOOA model portrayed a superior accuracy value of 97.16% over existing techniques.

## Data Availability

The raw data supporting the conclusions of this article will be made available by the authors, without undue reservation.
